# Membrane transport of root-borne *trans*-zeatin riboside maintains the cytokinin homeostasis in shoots

**DOI:** 10.1093/jxb/eraf369

**Published:** 2025-08-25

**Authors:** Daniel Nedvěd, Martin Hudeček, Petr Klíma, Jozef Lacek, Karel Müller, Anna Kuchařová, Petr Hošek, Ján Šmeringai, Markéta Pernisová, Václav Motyka, Ondřej Plíhal, Klára Hoyerová

**Affiliations:** Laboratory of Hormonal Regulations in Plants, Institute of Experimental Botany of the Czech Academy of Sciences, Rozvojová 263, Prague 165 00, Czechia; Department of Experimental Plant Biology, Faculty of Science, Charles University, Viničná 5, Prague 128 44, Czechia; Laboratory of Growth Regulators, Faculty of Science of Palacký University and Institute of Experimental Botany of the Czech Academy of Sciences, Šlechtitelů 27, Olomouc CZ-77900, Czechia; Laboratory of Hormonal Regulations in Plants, Institute of Experimental Botany of the Czech Academy of Sciences, Rozvojová 263, Prague 165 00, Czechia; Laboratory of Hormonal Regulations in Plants, Institute of Experimental Botany of the Czech Academy of Sciences, Rozvojová 263, Prague 165 00, Czechia; Laboratory of Hormonal Regulations in Plants, Institute of Experimental Botany of the Czech Academy of Sciences, Rozvojová 263, Prague 165 00, Czechia; Laboratory of Growth Regulators, Faculty of Science of Palacký University and Institute of Experimental Botany of the Czech Academy of Sciences, Šlechtitelů 27, Olomouc CZ-77900, Czechia; Laboratory of Hormonal Regulations in Plants, Institute of Experimental Botany of the Czech Academy of Sciences, Rozvojová 263, Prague 165 00, Czechia; Laboratory of Functional Genomics and Proteomics, National Centre for Biomolecular Research, Faculty of Science, Masaryk University, Brno 611 37, Czechia; Mendel Centre for Plant Genomics and Proteomics, Central European Institute of Technology, Masaryk University, Brno 625 00, Czechia; Laboratory of Functional Genomics and Proteomics, National Centre for Biomolecular Research, Faculty of Science, Masaryk University, Brno 611 37, Czechia; Mendel Centre for Plant Genomics and Proteomics, Central European Institute of Technology, Masaryk University, Brno 625 00, Czechia; Laboratory of Hormonal Regulations in Plants, Institute of Experimental Botany of the Czech Academy of Sciences, Rozvojová 263, Prague 165 00, Czechia; Laboratory of Growth Regulators, Faculty of Science of Palacký University and Institute of Experimental Botany of the Czech Academy of Sciences, Šlechtitelů 27, Olomouc CZ-77900, Czechia; Laboratory of Hormonal Regulations in Plants, Institute of Experimental Botany of the Czech Academy of Sciences, Rozvojová 263, Prague 165 00, Czechia; University of Maryland, USA

**Keywords:** Cytokinin, cytokinin riboside, cytokinin transport, ENT3, equilibrative nucleoside transporter, membrane transport

## Abstract

Ribosylated forms of the plant hormones cytokinins (CKs) are the dominant CK species translocated over long distances. The irreplaceable role of root-to-shoot translocated *trans*-zeatin riboside in the mediation of shoot development implies the existence of a yet-uncharacterized CK riboside-specific membrane transport system. In this work, we report significant differences in the kinetics of the membrane transport of CK nucleobases and ribosides and the overall affinity of membrane-bound carriers towards the two CK forms. We further characterize the membrane transport of CK nucleobases and ribosides mediated by Arabidopsis EQULIBRATIVE NUCLEOSIDE TRANSPORTER 3 (AtENT3) in tobacco BY-2 cells. Combining experimental data with computational modelling, we show that residues Tyr61 and Asp129, which are conserved among plant ENTs but not among ENTs from other species, are necessary for CK binding and that their mutation abolishes the ability of AtENT3 to transport CKs. Finally, we show that changes in *AtENT3* have different effects on the concentrations of *trans*-zeatin riboside throughout Arabidopsis plants and on the overall CK concentrations in roots, implying that AtENT3 participates in both the long- and the short-distance transport of CKs.

## Teaser

Cytokinin ribosides are transported by a distinct set of membrane-bound carriers, including EQUILIBRATIVE NUCLEOSIDE TRANSPORTER 3, whose transport function maintains cytokinin homeostasis in Arabidopsis shoots.

## Introduction

Cytokinins (CKs) are plant hormones that regulate a great variety of physiological processes, including cell cycle and proliferation (Miller *et al.*, 1956; Schaller *et al.*, 2014), growth and branching of both shoots and roots (Skoog and Miller, 1957; Werner *et al.*, 2001; Dello Ioio *et al.*, 2012; Schaller *et al.*, 2014; Chang *et al.*, 2015), chlorophyll retention and delay of senescence (Richmond and Lang, 1957; Dobránszki and Mendler-Drienyovszki, 2014; Talla *et al.*, 2016), and differentiation of vascular elements (Mähönen *et al.*, 2006; Bishopp *et al.*, 2011; De Rybel *et al.*, 2014). As signalling molecules, CKs participate in communication between various parts of the plant. They are distributed among tissues and organs through the two vascular pathways—phloem and xylem—but the CK composition in each of these pathways differs, and so presumably do their roles (Corbesier *et al.*, 2003; Hirose *et al.*, 2008; Osugi *et al.*, 2017; Sakakibara, 2021). To reach their eventual destination, CKs have to pass through biological membranes. One possible means of membrane transport is simple diffusion, which is described by Fick's laws (Paul *et al.*, 2014). Due to the hydrophobic character of the inner leaflets of the biological membrane, only small and non-polar molecules can cross the membrane this way. The required characterization applies to CK nucleobases, *N*^6^-substituted derivatives of adenine, which are the biologically active CK form (Lomin *et al.*, 2015). In contrast, CK ribosides, *N*^9^*-*ribosylated conjugates of CK nucleobases and the dominant CK components found in the vasculature (Corbesier *et al.*, 2003; Sakakibara, 2021), are bulky and polar, which implies that their diffusion would be inefficient (for a more detailed comparison, see Nedvěd *et al*., 2021). CK nucleobases and ribosides are recognized by membrane-bound carriers, which significantly improves the kinetics of their membrane transport. These carriers belong to the families of PURINE PERMEASES (PUPs) (Qi and Xiong, 2013; Zürcher *et al.*, 2016; Xiao *et al.*, 2019, 2020; Hu and Shani, 2023; Rong *et al.*, 2024), ATP-BINDING CASSETTES (ABCs) (Ko *et al.*, 2014; Zhang *et al.*, 2014; Zhao *et al.*, 2019, 2023; Kim *et al.*, 2020; Jarzyniak *et al.*, 2021; Yang *et al.*, 2022; Jamruszka *et al.*, 2024, Preprint), AZA-GUANINE RESISTANT (AZG) (Tessi *et al.*, 2021, 2023), SUGAR WILL EVENTUALLY BE EXPORTED TRANSPORTERS (SWEETs) (Radchuk *et al.*, 2023), and EQUILIBRATIVE NUCLEOSIDE TRANSPORTERS (ENTs) (Hirose *et al.*, 2005, 2008; Sun *et al.*, 2005; Girke *et al.*, 2014; Korobova *et al.*, 2021).

Given that CK ribosides are the main form of CKs transported over long distances, the membrane transport of ribosylated CKs represents a link between the long-distance and cell-to-cell CK distribution. Unlike CK nucleobases, CK ribosides can travel from the root up to the shoot apex and regulate processes such as leaf emergence rate in response to nutrient availability, which likely requires involvement of CK riboside transporters (Davière and Achard, 2013; Osugi *et al.*, 2017; Landrein *et al.*, 2018; Lopes *et al.*, 2021; Sakakibara, 2021).

The physiological importance of CK ribosides implies the existence of a CK riboside-specific system of membrane-bound carriers, possibly independent of the transport of CK nucleobases. Apparent candidates for these carriers are some members of the ENT family mentioned above. AtENT3, 6, and 8 from Arabidopsis have been characterized as CK transporters (Sun *et al.*, 2005; Hirose *et al.*, 2008; Korobova *et al.*, 2021). OsENT2 from rice (*Oryza sativa*, L.) has been directly shown to transport CKs across the biological membrane in a yeast model system (Hirose *et al.*, 2005), but whether OsENT2 acts as a CK transporter in plants remains to be answered.

In this work, we emphasize the importance of CK riboside transport by pinpointing the different kinetics of CK nucleobase and riboside uptake in the BY-2 cell line (*Nicotiana tabacum*, L. cv Bright Yellow 2), a plant single-cell system (Nagata *et al.*, 1992). Furthermore, we directly monitor AtENT3-mediated CK influx in BY-2, model interactions between AtENT3 and *trans*-zeatin riboside, and demonstrate the involvement of AtENT3 in shoot development in Arabidopsis.

## Materials and methods

### Plant material

We maintained tobacco BY-2 (*N. tabacum* L. cv Bright Yellow 2) cell lines in liquid Murashige and Skoog (MS) medium (30 g l^−1^ sucrose, 4.34 g l^−1^ MS salts, 100 mg l^−1^  *myo*-inositol, 1 mg l^−1^ thiamine, 0.2 mg l^−1^ 2,4-dichlorophenoxyacetic acid, 200 mg l^−1^ KH_2_PO_4_; pH 5.8), in the dark, at 27 °C, under continuous shaking (150 rpm; orbital diameter 30 mm), and subcultured it every 7 d. We cultured the *AtENT3*-expressing transgenic BY-2 cells and calli in the same medium supplemented with 100 mg ml^−1^ cefotaxime and 20 mg ml^−1^ hygromycin (indicating the final concentrations in media).

We grew the Arabidopsis ecotype Columbia 0 (Col-0), *atent3* T-DNA insertion mutant obtained from Nottingham Arabidopsis Stock Centre (under the accession of N631585), and *AtENT3* native overexpressor (NOE) on solid ^1^/_2_ MS medium (2.17 g l^−1^ MS salts, 10 g l^−1^ agar; pH 5.7) in Petri dishes and on Klasmann TS-3 fine cultivation substrate (Klasmann-Deilmann GmbH, Germany) in 7.0×7.0×6.5 cm pots. We kept the seeds sown on solid MS medium in the darkness at 4 °C for 3 d and then cultivated them for 8 d under long-day conditions (16 h light/8 h dark) at 20/22 °C in the D-root system (Silva-Navas *et al.*, 2015) using poly klima^®^ climatic growth chambers (poly klima GmbH, Germany). We randomly arranged the potted plants of different genotypes in transportable trays with a capacity of 20 pots (4×5 template) and grew them in cultivation chambers—phytotrons (CLF Plant Climatics, Germany) under long-day conditions at 21 °C with a LED light intensity of 130 μmol m^−2^ s^−1^ and 40–60% relative humidity. Unless stated otherwise, we obtained all chemicals and kits from Sigma-Aldrich.

### Preparation of estradiol-inducible AtENT3 constructs and transformation of BY-2 cells

To generate estradiol-inducible *AtENT3:GFP* and *AtENT3:Y61S:D129S:GFP* constructs for use in BY-2 transport assays, we employed a site-directed mutagenesis strategy based on NEBuilder^®^ HiFi DNA Assembly, using the wild-type *AtENT3* with C-terminal *GFP* fusion in the pENTR2B vector as a template. The wild-type *AtENT3:GFP* entry clone was first constructed by amplifying the genomic sequence of *AtENT3* using primers ENT3_SalI_FW and ENT3+6_NotI_RE, introducing *Sal*I and *Not*I restriction sites. The PCR product was cloned into the *pENTR2B_GFP* vector (Králová *et al.*, 2024) via *Sal*I/*Not*I digestion and ligation, resulting in *AtENT3:GFP* in the *pENTR2B* vector. Site-directed mutagenesis was performed to introduce two point mutations: Y61S and D129S. The mutations were introduced via two-fragment NEBuilder^®^ HiFi DNA Assembly using primer pairs SD_ENT3_Y61S_FW/SD_ENT3_Y61S_RE and SD_ENT3_D129S_FW/SD_ENT3_D129S_RE, respectively. The resulting construct, *AtENT3:Y61S:D129S:GFP*, was maintained in the *pENTR2B* backbone. Finally, this entry clone was recombined with the estradiol-inducible destination vector *pMDC7* (Curtis and Grossniklaus, 2003) using Gateway^®^ LR Clonase^®^ II, generating the final construct *AtENT3:Y61S:D129S:GFP* in *pMDC7*, suitable for inducible expression in BY-2 cells. The BY-2 cells were transformed by co-cultivation with *Agrobacterium tumefaciens* strain GV2260 (An *et al.*, 1985). The transgenic lines were harvested after 4 weeks, cultured on solid media with kanamycin, and tested for the presence of *AtENT3* via PCR.

### Preparation of the *pAtENT3::GFP:ENT3:3′-UTR* construct for the generation of native *AtENT3*-overexpressing Arabidopsis plants

We employed a multi-step cloning strategy to generate a construct for *GFP:AtENT3* expression under the control of the native *AtENT3* promoter. First, the vector *pENTR2B_GFP2* was generated to allow the N-terminal green fluorescent protein (GFP) fusion. The *GFP* coding sequence was PCR-amplified from the existing *pENTR2B_GFP* (Králová *et al*., 2024) vector using primers GFP_SalI_FW01 and GFP_NotI_KpnI_XhoI_RE01, introducing *Sal*I and *Xho*I restriction sites. The amplified fragment replaced the original C-terminal GFP in *pENTR2B_GFP* via *Sal*I/*Xho*I digestion and ligation, resulting in *pENTR2B_GFP2*, a vector suitable for N-terminal GFP fusions. Next, the *AtENT3* genomic fragment (excluding the promoter) was amplified from genomic DNA using primers ENT3_GGGGS_NotI_FW01 and ENT3+6_XhoI_RE01, introducing a flexible GGGGS linker and compatible *Not*I/*Xho*I restriction sites. This fragment was inserted into *pENTR2B_GFP2* via *Not*I/*Xho*I, placing *AtENT3* in-frame downstream of *GFP*, yielding the intermediate *GFP:AtENT3* construct in the *pENTR2B* backbone. To include native regulatory elements, the 4 kb region upstream of the *AtENT3* start codon was amplified using primers GA_ENT3_4kb_FW and GA_ENT3_4kb_RE. The *GFP:AtENT3* fragment was re-amplified using GA_ENT3_Nterm_pENTR2b_FW and GA_ENT3_Nterm_pENTR2b_RE, and both fragments were assembled using NEBuilder^®^ HiFi DNA Assembly, resulting in the *pAtENT3::GFP:AtENT3* construct in *pENTR2B*. Subsequently, the 3′-untranslated region (UTR) of *AtENT3* was amplified using GA_ENT3_3UTR_FW and GA_ENT3_3UTR_RE. The *pAtENT3::GFP:AtENT3 pENTR2B* construct was re-amplified with GA_ENT3_4kb_Nterm_pENTR2b_FW and GA_ENT3_4kb_Nterm_pENTR2b_RE, and both fragments were assembled using NEBuilder^®^ HiFi DNA Assembly, yielding the final *pAtENT3::GFP:AtENT3:3′-UTR* construct in the *pENTR2B* vector. Finally, this entry clone was recombined with the destination vector *pMCS:GW* (Michniewicz *et al.*, 2015) via the Gateway^®^ LR Clonase^®^ II reaction, resulting in the expression construct *pAtENT3::GFP:AtENT3:3′-UTR* in *pMCS:GW*, suitable for plant transformation. We generated the *AtENT3* NOE lines by introducing the expression vector into the Col-8 background using the floral dip method (Clough and Bent, 1998) with *A. tumefaciens* strain GV3101. Primary transformants (T₁ generation) were selected on soil by spraying with 0.02% Basta^®^ (glufosinate-ammonium; Bayer, Germany). Subsequent generations were selected on MS medium supplemented with 15 mg l^−1^ phosphinothricin.

### Radio-accumulation assays

For the radio-accumulation assays, we used BY-2 cell suspensions 2 d after inoculation. We filtered away the liquid phase of the suspension, twice resuspended the cells in uptake buffer (20 mM MES, 10 mM sucrose, 0.5 mM CaSO_4_, pH 5.7), and cultivated them in the dark for 45 and 90 min, respectively. The assay itself was initiated by applying a radio-labelled tracer (provided by the Isotope Laboratory at the Institute of Experimental Botany, Prague, Czechia) into the cell suspension and terminated after 15–30 min. During the assay, we sampled 500 µl of the suspensions in regular intervals. For each sample, we filtered away the liquid phase and treated the cells with 500 µl of 96% (v/v) ethanol for 30 min. Next, we added 4 ml of scintillation cocktail EcoLite(+)™ (MP Biomedicals, CA, USA) to each sample and mixed the samples for 20 min using orbital shaker KS 130 (IKA, Germany) at 480 rpm. The radioactivity in samples was measured using a Tri-Carb 2900TR scintillation counter (PerkinElmer, CT, USA).

### Mathematical modelling of transport kinetics

To describe the kinetics of CK membrane transport in BY-2 cell culture, we adapted the model published by Hošek *et al*. (2012). We introduced first-order rate constants *I* and *E* to characterize the influx and efflux of a radio-labelled tracer, respectively. To account for the tracer adsorption to the cell surfaces, we included a factor *K*. To estimate the values of *I*, *E*, and *K*, we fitted experimental data from radio-accumulation assays with the equation:


(1) $$c_{I}(t)=\frac{Ic_{0}}{\;fI+E}\left[1-e^{-t(\;fI+E)}\right](1-fK)+Kc_{0},$$

where *t* and *c*_I_ are matrices of time points and measured intracellular concentrations, respectively (with each row composed of data points from one assay and different rows representing different assays), *f* is a factor correcting different sizes of the intra- and extracellular spaces, and *c*_0_ is the initial extracellular concentration of the tracer. When comparing the effects of the *AtENT3* expression or a chemical treatment on the tracer influx, we constrained the model to keep common values of *E* and *K* for all assays in the dataset. For assays involving chemical treatment during the tracer accumulation (as opposed to the treatment before the tracer addition), we used an expanded form of Equation (1):


(2) $$\begin{array}{rl} & c_{I}(t)=Kc_{0}+(1-fK)\\ & \{\begin{array}{ccc} \frac{Ic_{0}}{\;fI+E}\left[1-e^{-t(\;fI+E)}\right] & \mathrm{if} & t\leq t^{\prime }\\ \frac{I^{\prime }c_{0}}{\;fI^{\prime }+E}\left[1-e^{\left(t^{\prime }-t)(\;fI^{\prime }+E\right)}\right]+\frac{Ic_{0}}{\;fI+E}\left[1-e^{-t^{\prime }(\;fI+E)}\right]e^{\left(t^{\prime }-t)(\;fI^{\prime }+E\right)} & \mathrm{if} & t>t^{\prime } \end{array}, \end{array}$$

Where *t*′ is the treatment time and *I*′ is the influx rate constant after the treatment.

To evaluate the affinity of the membrane transport system towards a tracer or the inhibitory effect of a competitor, we adapted a saturation model published by Delbarre *et al*. (1996):


(3) $$I(c_{K})=\frac{v_{\lim}}{IC_{50}+c_{K}}+D,$$

where *c*_K_ is the concentration of a competitor (either the non-labelled counterpart of the tracer or another chemical substance), *v*_lim_ is the limit transport rate, IC_50_ is the *c*_K_ value for which the transport rate is equal to half *v*_lim_, and *D* is the rate constant of the influx that remains even when the transport system is fully saturated.

All fits were performed using the ‘curve_fit’ method of the SciPy Python library (Virtanen *et al.*, 2020) with arguments ‘ftol=1e-15’ and ‘xtol=1e-15’. The initial guesses were 10^−3^ for *I* and *E*, 0 for *K*, and 1 for *v*_lim_, IC_50_, and *D*. All parameters were restricted to be non-negative. To visualize tracer accumulation in the cells, we used Equation (1) with the optimized values of *I* and *E,* while setting *K* to 0.

### Molecular docking

For molecular docking, we downloaded AlphaFold-predicted structural models (Jumper *et al.*, 2021) from the AlphaFold Protein Structure Database (https://alphafold.ebi.ac.uk/). We prepared the protein and ligand files and performed the docking procedure using the AutoDockFR (ADFR) software suite (Zhao *et al.*, 2006; Ravindranath *et al.*, 2015). The ligands were initially placed in the central cavity of the protein. The centre and dimension of the affinity grids were determined automatically by the ADFR program ‘agfr’. Each docking consisted of 50 runs and each run performed 50 million evaluations. Residues Gln133, Arg312, Leu397, and Asp129 of AtENT3 were set flexible. To visualize the protein–ligand structures, we used ChimeraX (Goddard *et al.*, 2018), PyMol (Schrödinger, LLC, 2015), and LigPlot+ (Laskowski and Swindells, 2011) software. We used MAFFT with the automatic algorithm selection (Katoh and Standley, 2013) to align the ENT sequences and JalView to visualize their alignment (Waterhouse *et al.*, 2009).

### Molecular dynamics

For the molecular dynamic simulations, we built a rhombic dodecahedron-shaped simulation box consisting of the protein–ligand complex in 150 mM aqueous NaCl solution and additional ions to neutralize the electric charge. To prevent the complex from interacting with its own image, we set its distance from the box edges to 1.0 nm. We ran energy minimization using the steepest descent algorithm, a 100 ps-long simulation under the *NVT* ensemble (constant particle amount, volume, and temperature), a 100 ps-long simulation under the *NPT* ensemble (constant particle amount, pressure, and temperature), and finally 100 ns or 200 ns-long unbiased simulation. In the *NVT* and *NPT* runs, we applied constraints with force constants of 1000 kJ mol^−1^ nm^2^ to all non-hydrogen atoms. For all runs, we used the CHARMM36 force field (Best *et al.*, 2012). Simulation parameters are given in Supplementary Table S1. We used the GROMACS software suite (Abraham *et al.*, 2015, 2024; Páll *et al.*, 2015) to parametrize the protein, build the simulation box, carry out the simulations, perform the cluster analysis, and calculate the distributions of distances over the trajectories. Through GROMACS, we also used the particle mesh Ewald method to evaluate long-range interactions (Essmann *et al.*, 1995), the LINCS algorithm to solve constraints (Hess, 2008), and the SETTLE algorithm to treat water molecules (Miyamoto and Kollman, 1992). To parametrize the ligand, we used the CGenFF program (Vanommeslaeghe and MacKerell, 2012; Vanommeslaeghe *et al.*, 2012). To calculate fractional occupancies of the system by water molecules, we used VMD software (Humphrey *et al.*, 1996).

### Plant phenotyping and imaging

For phenotyping of 8-day-old Arabidopsis plants grown on agar, we isolated their shoots, placed these shoots on Petri dishes, and scanned them from a top view using Epson Perfection V700 Photo (Epson, Japan). To image the potted plants, we used the PlantScreen™ Compact System (PSI, Czechia) equipped with PSI DUAL camera containing two 12.36 megapixel CMOS sensors: a colour Sony IMX253LQR-C sensor for RGB structural imaging and a monochromatic Sony IMX253LLR-C for chlorophyll fluorescence measurement (Sony, Japan). For the fluorescence measurement, we used a quenching analysis protocol. Raw data were automatically processed using the PlantScreen™ Analyzer software (PSI). The imaging was performed according to a previously published protocol (Šmeringai *et al.*, 2023, Preprint). Shoots of 4-day-old *AtENT3* NOE seedlings were imaged using the widefield mode of the Zeiss LSM900 microscope with the Plan-Apochromat ×10/0.45 NA M27 objective and Axiocam 712 camera (Zeiss, Germany). We detected GFP fluorescence with the LED-Module at 475 nm at 20% intensity (150 ms exposure) and chlorophyll autofluorescence with the LED-Module at 630 nm at 1.1% intensity (76 ms exposure).

### Image processing

We transformed the images of shoots isolated from the agar-grown plants from the RGB to the L*a*b* space and segmented the shoots by applying the thresholds a*≤−9.5, b*≥−9.5, and L*≥18.5, based on estimates obtained by the multi-Otsu method (Liao *et al.*, 2001). In the binary mask, we removed all objects smaller than 2048 pixels, performed morphological closing using a disk-shaped footprint with a radius of 8 pixels, and then removed all objects smaller than 8192 pixels. Finally, we measured the areas of all remaining objects in the image. To implement the techniques listed above, we used the Python scikit-image library (van der Walt *et al.*, 2014). We processed images of potted plants according to Šmeringai *et al*. (2023, Preprint). For miscellaneous image manipulations, we used the GNU Image Manipulation Program (The GIMP Team, 2024).

### Reverse transcription–quantitative PCR

We isolated total RNA from plant shoots using the RNeasy Plant Mini kit (Qiagen, Germany) and treated it with the DNA-Free Kit (Thermo Fisher Scientific, MA, USA). We evaluated the purity, concentration, and integrity of RNA on 0.8% agarose gels (v/w) and with the RNA Nano 6000 Assay Kit using the Bioanalyzer instrument (Agilent Technologies, CA, USA). For reverse transcription of approximately 1 mg of the DNAse-treated RNA, we used M-MLV Reverse Transcriptase, RNase H(−), Point Mutant (Promega, WI, USA). We performed the quantitative PCR using GoTaq qPCR Master Mix (Promega) at the annealing temperature of 58 °C on the LightCycler480 instrument (Roche, Switzerland). PCR efficiency was estimated using serial dilution of template cDNA. We calculated the relative expression level (REL) as follows:


(4) $$\mathrm{REL}=\frac{\sqrt{2^{CP_{R1}+CP_{R2}}}}{2^{\mathrm{CP}}},$$

where CP_R1_ and CP_R2_ are the crossing points of two reference genes, and CP is the crossing point of the target gene. We used Arabidopsis elongation factor 1a (*AtEF1a*) and actin 2 (*AtACT2*) as reference genes. We verified positive transcript levels and the quality of PCR by the melting curve analysis. The primer sequences are listed in Supplementary Table S2.

### Determination of cytokinins in plant tissues

We separated roots, hypocotyls, cotyledons, and shoot apices (the remaining tissue) of 8-day-old seedlings using microscissors. We took 5–20 mg per one sample of one tissue. We applied 1 M formic acid to collected tissue samples and homogenized the mixture using the FastPrep-24 grinder (MP Biomedicals, USA). We centrifuged the homogenates at 30 130*×g* and 4 °C for 25 min using the 5430R centrifuge (Eppendorf, Germany). We collected the supernatant, resuspended the pellet in 1 M formic acid, and repeated the centrifugation as described. We applied the combined supernatant to the Oasis HLB 96-well µElution plates (Waters, USA) that we had previously activated with acetonitrile p.a., ultrapure water, and 1 M formic acid. We washed the plate wells with ultrapure water and eluted the CK-containing fraction with 50% acetonitrile, using the Pressure+vacuum manifold (Biotage, Sweden). We analysed the contents of eluted fractions using the 1290 Infinity II ultra-high pressure liquid chromatograph (Agilent, CA, USA) in tandem with the 6495 triple quadrupole liquid chromatography–mass spectrometry system (Agilent).

### Metabolic profiling of radio-labelled compounds

To determine the purity of used radio-labelled tracers, as well as their metabolic conversions in BY-2 cells, we analysed metabolic spectra of stored tracers and samples of cells treated with 20 nM tracers taken at time points 1, 5, 10, and 15 min. We isolated and purified CK fractions from the cell samples according to Dobrev and Kamínek (2002). Stored tracer samples and purified CK fractions were subject to HPLC. The HPLC equipment consisted of the Series 200 autosampler and quaternary pump (PerkinElmer), Luna C18(2) column (Phenomenex, CA, USA) heated at 35 °C, and two detectors coupled in series: the 235C diode array detector (PerkinElmer) and Ramona 2000 flow-through radioactivity detector (Raytest, Germany). Two solvents (A: 40 mM CH_3_COOH adjusted with NH_4_OH to pH 4.0 and B: CH_3_CN/CH_3_OH, 1/1, v/v) were used at a flow rate of 0.6 ml min^−1^. The column eluate was monitored at 270 nm by the diode array detector, then on-line mixed with a 3-fold volume of the liquid scintillation cocktail Flo-Scint III (Packard BioScience Co., CT, USA) and monitored by the Ramona 2000 radioactivity detector. The metabolites of radio-labelled CKs were identified by comparing their retention times with authentic standards.

### Confocal imaging of BY-2 cells

*In vivo* confocal microscopy of BY-2 cells was performed using the Zeiss LSM 880 confocal microscope with Airyscan and the Plan-Apochromat ×63/1.4 Oil DIC M27 objective. The cells were incubated with the membrane-probing dye FM 4-64 (Thermo Fisher Scientific) (2 µM, 20 min). The following fluorescence excitation and emission settings were used to visualize the reporter genes and staining signals: GFP excitation 488 nm, emission 516 nm; FM 4-64 excitation 561 nm, emission 579 nm. Sequential scanning was used for the co-localization studies to avoid interference between the fluorescence channels. The images were processed with the image analysis software Zeiss Zen 2.5 (blue edition) and compiled for presentation in Adobe Photoshop.

## Results

### Transport of cytokinin ribosides in BY-2 cells occurs with kinetics distinct from cytokinin nucleobases and depends on proton gradient

To find out whether the membrane transport kinetics of ribosylated CKs differ from the kinetics of CK nucleobases, the biologically active CK form, we measured the uptake of various radio-labelled CK tracers in tobacco BY-2 cell cultures (Nagata *et al.*, 1992), a model plant single-cell system. The radio-labelled tracers comprised four CK nucleobases, [^3^H]*trans*-zeatin (tZ), [^3^H]dihydrozeatin (DHZ), [^3^H]isopentenyladenine (iP), and [^3^H]benzyladenine (BA), and four CK ribosides, [^3^H]*trans*-zeatin riboside (tZR), [^3^H]dihydrozeatin riboside (DHZR), [^3^H]isopentenyladenosine (iPR), and [^3^H]benzyladenosine (BAR). With this selection, we also included CKs with diverse character of their side chains (*N*^9^-bound moieties), namely those with unsaturated hydroxylated chains (tZ, tZR), saturated hydroxylated chains (DHZ, DHZR), unsaturated aliphatic chains (iP, iPR), and aromatic chains (BA, BAR).

**Fig. 1.**
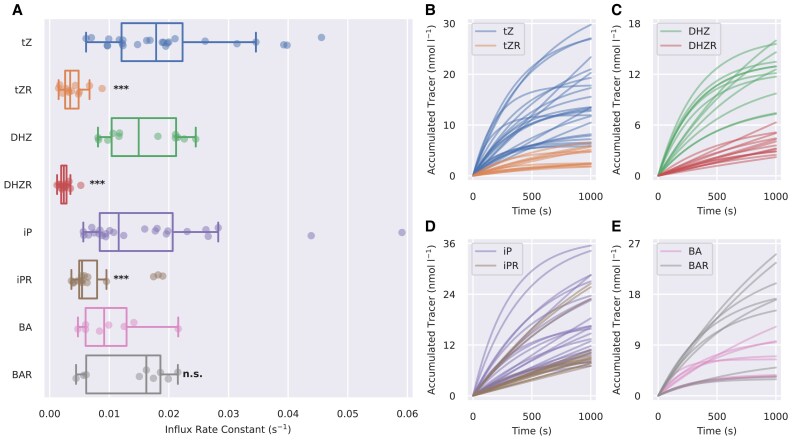
Characterization of CK membrane transport in tobacco BY-2 cells. (A) Estimated values of the influx rate constant (*I*) for different radio-labelled CK tracers obtained by fitting data from radio-accumulation assays to Equation (1). (B–E) Comparison of the accumulation trends (concentration of the accumulated tracer over time) between CK nucleobases and their ribosylated forms. The curves are aligned by setting *K* = 0 and *c*_0_=2 nM for each assay. *P*-values obtained from the Kruskal–Wallis test comparing *I*-values for the corresponding pairs of CK nucleobases and ribosides: n.s., *P*≥0.05; ***P<0.001. BA, benzyladenine; BAR, benzyladenosine; DHZ, dihydrozeatin; DHZR, dihydrozeatin riboside; iP, isopentenyladenine; iPR, isopentenyladenosine; tZ, *trans*-zeatin; tZR, *trans*-zeatin riboside.

We used the mathematical model given by Equation (1) to obtain parameters of the membrane transport kinetics for each tracer. We focused on the parameter *I*, the import rate constant, which approximates the total transport rate at the beginning of the assay. The median *I*-values of tZ (17.86×10^−3^ s^−1^), DHZ (14.94×10^−3^ s^−1^), and iP (11.58×10^−3^ s^−1^) were greater than those of tZR (3.45×10^−3^ s^−1^), DHZR (2.38×10^−3^ s^−1^), and iPR (5.44×10^−3^ s^−1^). This result shows that BY-2 cells accumulate CK nucleobases with non-aromatic side chains more readily than their respective ribosides. Regarding the side chain composition, the differences in transport among tZ-type, DHZ-type, and iP-type CKs were less pronounced than the differences between CK nucleobases and ribosides. The median values of *I* obtained for BA (9.14×10^−3^ s^−1^) and BAR (16.22×10^−3^) showed the opposite trend to isoprenoid CKs—the riboside was, in this case, transported more readily than the nucleobase. The accumulation trends and *I*-values of all tracers are depicted in Fig. 1. The complete list of kinetic parameters and their statistical analysis are presented in Supplementary Tables S3 and S4, respectively. The observed transport kinetics can be affected by tracer degradation during storage or their metabolic conversions in BY-2 cells. To address these effects, we measured the purity of tracers and their metabolic profiles in BY-2 cells. While the purities of all used tracers were above 80% (Supplementary Table S5), their metabolic conversions occurred quite rapidly during the transport assay (Supplementary Table S6). Use of the parameter *I*, which approximates the initial transport kinetics, can partially counter the effect of tracer metabolic conversions.

**Fig. 2.**
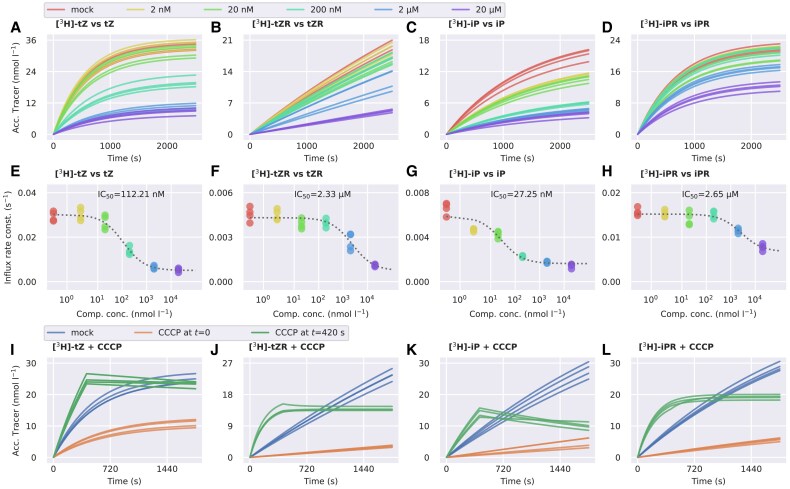
Saturation of the CK membrane transport in tobacco BY-2 cells. (A–D) Accumulation trends (concentration of the accumulated tracer over time) of radio-labelled CK in BY-2 cells inhibited by increasing concentrations of their non-labelled counterparts. The shape of the curves is determined by the *I* and *E* values obtained by fitting the data from radio-accumulation assays to Equation (1). For the visualization purposes, *K* is set to 0 and *c*_0_ to 2 nM for each assay. (E–H) Dependence of *I* values obtained from the mathematical modelling of radio-accumulation data on the concentration of the non-labelled competitors. The plotted data points are further fitted to Equation (3) to obtain the saturation parameters. The fit of Equation (3) is represented by grey dashed curves. The *I*-values correspond to the curves depicted in (A–D). (I–L) Accumulation trends of radio-labelled CK tracers in presence of 50 µM carbonyl cyanide 3-chlorophenylhydrazone (CCCP). Acc., accumulated; iP, isopentenyladenine; iPR, isopentenyladenosine; tZ, *trans*-zeatin; tZR, *trans*-zeatin riboside.

To confirm that the observed uptake of CK nucleobases and ribosides occurs by carrier-mediated transport, we performed a series of assays in which we accumulated [^3^H]tZ, [^3^H]tZR, [^3^H]iP, or [^3^H]iPR together with their non-labelled counterparts (so-called competitors) at concentrations of 0, 2 nM, 20 nM, 200 nM, 2 µM, and 20 µM. Each dataset, consisting of experiments performed with one tracer and all concentrations of the corresponding non-labelled competitor, was fitted to the constrained variant of Equation (1), i.e. with single values of *E* and *K* for the whole dataset. The uptake of CK nucleobases and ribosides was subject to dose-dependent inhibition by their non-labelled variant, indicating that their transport is mediated by saturable membrane-bound carriers (Fig. 2A–D). To evaluate this inhibition effect numerically, we fitted the *I*-parameter values obtained from the assays with competitors using the saturation model given by Equation (3) (Delbarre *et al.*, 1996). The estimated IC_50_ values obtained for tZ (112.21 nM), tZR (2.33 µM), iP (27.25 nM), and iPR (2.65 µM) showed that CK nucleobases are transported with slightly higher affinity than the corresponding ribosides, which further indicates the distinct transport properties of the two CK forms (Fig. 2E–H). The complete list of kinetic parameters is presented in Supplementary Table S7.

To assess the thermodynamic aspect of the uptake of tZ, tZR, iP, and iPR in the BY-2 cells, we performed a series of accumulation assays in suspensions treated with 50 µM of the protonophore carbonyl cyanide 3-chlorophenylhydrazone (CCCP) in dimethyl sulfoxide (DMSO). CCCP uncouples electron transfer from oxidative phosphorylation (Heytler, 1963; Cavari *et al.*, 1967; Cunarro and Weiner, 1975), thus inhibiting proton gradient-dependent transport processes (Culos and Watanabe, 1983; Alexander *et al.*, 2018; Stoffer-Bittner *et al.*, 2018). We performed the same assays using cells treated with the corresponding amount of DMSO alone (mock treatment) as control. We fitted all data to Equation (1) to obtain *I*-values. The CCCP treatment decreased the medians of *I* (in comparison with the mock treatment) for all four tracers, from 24.24×10^−3^ to 8.89×10^−3^ s^−1^ for tZ, from 7.78×10^−3^ to 0.91×10^−3^ s^−1^ for tZR, from 9.89×10^−3^ to 1.46×10^−3^ for iP, and from 11.33×10^−3^ to 1.86×10^−3^ s^−1^ for iPR (Fig. 2I–L), indicating that the uptake of both CK nucleobases and ribosides at least partially occurs in a proton gradient-dependent manner. The complete list of kinetic parameters and their statistical analysis are presented in Supplementary Tables S8 and S9, respectively. To observe the immediate response of the CK influx to the uncoupling of the proton gradient, we performed another set of assays, in which we treated the cells with 50 µM CCCP in DMSO 7 min after adding the tracer. We fitted the measured data with Equation (2) to visualize the response (the estimated kinetic parameters are presented in Supplementary Table S10). The fits showed that after the treatment, the intracellular concentrations of CK nucleobases start to decrease, while the concentrations of CK ribosides stop increasing and remain constant (Fig. 2I–L). The former could indicate the presence of CCCP-resistant exporters of CK nucleobases.

### BY-2 cells possess a riboside-specific transport system that does not recognize cytokinin nucleobases as substrates

**Fig. 3.**
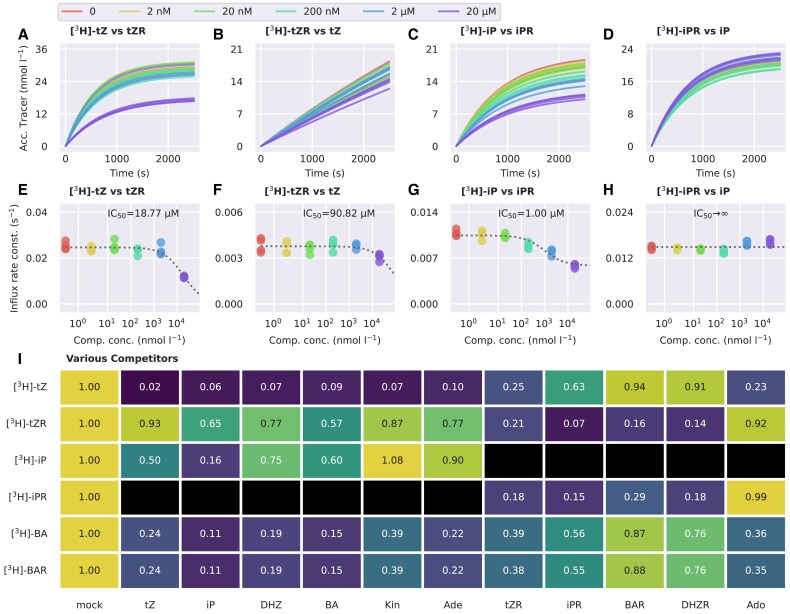
Substrate specificity of CK membrane-bound transport systems. (A–D) Accumulation trends (concentration of the accumulated tracer over time) of radio-labelled CK in BY-2 cells inhibited by increasing concentrations of chemically diverse non-labelled substances. The shape of the curves is determined by the *I-* and *E*-values obtained by fitting data from radio-accumulation assays to Equation (1). For visualization purposes, *K* is set to 0 and *c*_0_ to 2 nM for each assay. (E–H) Dependence of the *I*-values obtained from the mathematical modelling of radio-accumulation data on the concentration of the non-labelled competitors. The plotted data points are further fitted to Equation (2) to obtain the saturation parameters. The fit of Equation (2) is represented by grey dashed curves. The *I*-values correspond to the curves depicted in (A–D). (I) Fold changes of the influx rate constants estimated for various combinations of radio-labelled CK tracers and 20 µM non-labelled competitors. Black cells denote non-tested combinations. Acc., accumulated; Ade, adenine; Ado, adenosine; BA, benzyladenine; BAR, benzyladenosine; DHZ, dihydrozeatin; DHZR, dihydrozeatin riboside; iP, isopentenyladenine; iPR, isopentenyladenosine; Kin, kinetin; tZ, *trans*-zeatin; tZR, *trans*-zeatin riboside.

The different affinities of the BY-2 membrane-bound carriers towards CK nucleobases and ribosides (Fig. 2E–H) imply either that both CK types are recognized by the same set of carriers (with the nucleobases being slightly preferred) or that there are two sets of carriers, one for nucleobases and one for ribosides, that function independently of one another. To see which of these models characterized the CK transport in BY-2 cells better, we paired nucleobase tracers with riboside competitors and vice versa (i.e. [^3^H]tZ with tZR, [^3^H]tZR with tZ, [^3^H]iP with iPR, and [^3^H]iPR with iP) and repeated the radio-accumulation assays with increasing concentrations of non-labelled CKs. We fitted the experimental data to Equation (1) to assess kinetic parameters for each assay (Fig. 3A–D) and the obtained values of *I* to Equation (3) to estimate the IC_50_ for each competitor (Fig. 3E–H). The estimated IC_50_ values were 18.77 µM ([^3^H]tZ vs tZR), 90.82 µM ([^3^H]tZR vs tZ), and 1.00 µM ([^3^H]iP vs iPR). No saturation occured for the [^3^H]iPR vs iP variant (i.e. IC_50_ diverges towards infinity). The complete list of kinetic parameters is presented in Supplementary Table S5. These results show that the inhibition of CK nucleobase uptake by CK ribosides is significantly weaker than the inhibition by non-labelled nucleobases, and conversely, the inhibition of CK riboside uptake by non-labelled ribosides is stronger than the inhibition by nucleobases. This trend supports the hypothesis that CK nucleobases and ribosides are transported by at least two independent carrier sets.

To confirm this trend, we tested the inhibitory effect of more CK- and adenine-based competitors (adenine, tZ, iP, BA, DHZ, kinetin, adenosine, tZR, iPR, BAR, and DHZR) on the uptake of [^3^H]tZ, [^3^H]tZR, [^3^H]iP, [^3^H]iPR, [^3^H]BA, and [^3^H]BAR. The concentration of all competitors was 20 µM. We fitted the measured data to the constrained variant of Equation (1) and compared the median values of *I*. The uptake of [^3^H]tZ and [^3^H]BA decreased (two to four times) in the presence of all tested nucleobases, as well as tZR, adenosine, and iPR ([^3^H]BAR only), whereas other tested ribosides caused mild to no uptake inhibition. The uptake of [^3^H]iP was about three times reduced by its non-labelled variant but only mildly reduced by other tested compounds. The uptake of all three labelled ribosides was efficiently inhibited by all tested ribosides with a striking exception of adenosine and only mildly inhibited by tested nucleobases (Fig. 3I; for the estimated kinetic parameters, see Supplementary Table S11). The results presented so far indicate the existence of at least two systems mediating the CK membrane transport—one can recognize both CK nucleosides and ribosides (with a slight preference towards the former), while the other is strictly riboside-specific.

### AtENT3 transports cytokinin nucleobases and ribosides in tobacco BY-2 cell system

To see how our previous conclusions about the CK membrane transport as a whole apply to individual membrane-bound carriers, we went on to express a previously characterized transporter of CK ribosides in BY-2 cells. This way, we could measure the transporter's contribution to the uptake of tZ, iP, tZR, and iPR. We focused on members of the ENT family, as some of them have been linked to the transport of CK ribosides (Girke *et al.*, 2014).

**Fig. 4.**
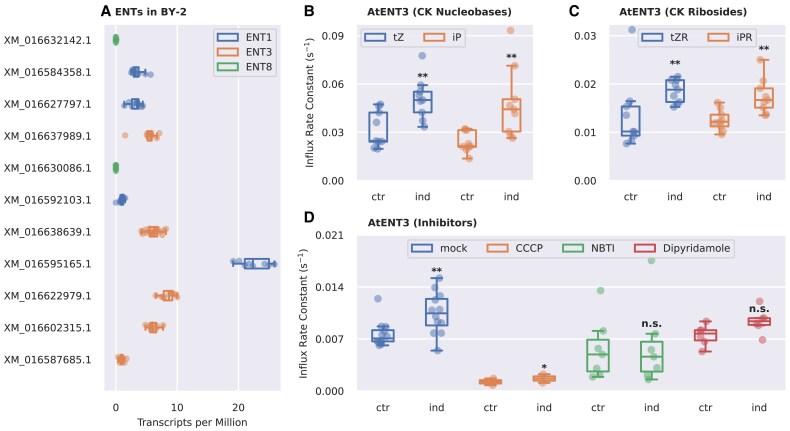
The effect of *AtENT3* expression on CK uptake in tobacco BY-2 cells. (A) Expression of *AtENT* homologues in 2-day-old BY-2 cultures. The identifiers on the vertical axis correspond to accessions in the NCBI Gene database (accessed on 17 April 2024). Data were obtained through the Gene Expression Omnibus (GEO) database under the accession GSE160438 (Müller *et al*., 2021). (B, C) Optimized values of the influx rate constant, *I*, obtained by fitting to Equation (1) data from radio-accumulation assays measuring the uptake of radio-labelled CK nucleobases and ribosides in the *AtENT3*-harbouring BY-2 cells under an estradiol-inducible promoter without (ctr) or with the induction (ind) of *AtENT3* expression. (D) Optimized values of *I* for the uptake of radio-labelled tZR in the *AtENT3*-harbouring BY-2 cells without or with the induction of *AtENT3* expression and without (mock) or with transport inhibitors *S*-(4-nitrobenzyl)-6-thioinosine (NBTI), dipyridamole (DiPy), and carbonyl cyanide 3-chlorophenylhydrazone (CCCP). All inhibitors were applied at a concentration of 10 µM. *P*-values obtained from the Kruskal–Wallis test comparing *I*-values for induced and control cell lines: n.s., *P*≥0.05; **P<0.01. CK, cytokinin; ENT, EQULIBRATIVE NUCLEOSIDE TRANSPORTER; iP, isopentenyladenine; iPR, isopentenyladenosine; tZ, *trans*-zeatin; tZR, *trans*-zeatin riboside.

To determine whether tobacco ENTs could be responsible for the CK uptake in BY-2 cells, we searched for expression of *ENT* genes in a previously published BY-2 transcriptome (Müller *et al.*, 2021). All tobacco ENTs listed in the UniProtKB database (The UniProt Consortium, 2023) are homologs of AtENT1, 3 or 8. Of these, only the homologs of *AtENT1* and *3* are expressed in BY-2 (Fig. 4A; numerical values are presented in Supplementary Table S12), implying that measurements performed on AtENT1 or 3 may reflect the transport trends described above. We eventually decided to use AtENT3, given the previous reports on its effects on the CK homeostasis and plant sensitivity to exogenously applied CKs (Sun *et al.*, 2005; Korobova *et al.*, 2021).

To directly monitor the transport activity of AtENT3 towards CKs, we introduced the estradiol-inducible *AtENT3:GFP* gene construct to BY-2 cells. We checked the expression of the construct microscopically (Supplementary Fig. S1). Using these transformed cells, we performed radio-accumulation assays with [^3^H]tZ, [^3^H]tZR, [^3^H]iP, and [^3^H]iPR as tracers. We performed each assay in non-induced (control) and induced cells to assess the contribution of AtENT3 to the overall CK uptake. The induced cells were treated with 1 µM estradiol in DMSO and the control cells with the corresponding amount of DMSO. The measured data were fitted to the constrained variant of Equation (1) to estimate *I*-values. The medians of *I* increased for all four tracers: from 24.56×10^−3^ to 50.03×10^−3^ s^−1^ for tZ, from 10.16×10^−3^ to 18.82×10^−3^ s^−1^ for tZR, from 21.34×10^−3^ to 44.27×10^−3^ s^−1^ for iP, and from 12.19×10^−3^ to 16.69×10^−3^ for iPR (Fig. 4B, 4C). The complete list of kinetic parameters and their statistical analysis is presented in Supplementary Tables S13 and S14, respectively. These results show that AtENT3 transports nucleobases and ribosides, implying that AtENT3 is likely not a part of the previously described CK riboside-specific system and that strict carriers of ribosylated CKs remain to be identified.

To further confirm that AtENT3 is responsible for the increase in CK riboside uptake in the induced cells, we examined how AtENT3-mediated uptake of tZR changes after application of CCCP and two inhibitors of nucleoside uptake, 4-nitrobenzylthioinosine (NBTI) (Ward *et al.*, 2000; Wright and Lee, 2019; Karbanova *et al.*, 2020) and dipyridamole (DiPy) (Newell *et al.*, 1986; Woffendin and Plagemann, 1987). NBTI and DiPy inhibit the uptake of adenosine by AtENTs (Möhlmann *et al.*, 2001; Li *et al.*, 2003; Wormit *et al.*, 2004). To assess the effects of the inhibitors, we performed accumulation assays with [^3^H]tZR as a substrate, in non-induced (control) or induced cell lines and with or without CCCP, NBTI, or DiPy. All inhibitors were dissolved in DMSO and used at the concentration of 10 µM. For mock treatment, we used the corresponding amount of DMSO alone. We fitted the measured data to the constrained variant of Equation (1). In mock-treated cells the uptake of tZR significantly increased (from 7.56×10^−3^ to 10.87×10^−3^ s^−1^) due to the induction of *AtENT3* expression. In CCCP-treated cells, the overall tZR uptake dropped regardless of the *AtENT3* expression status. The difference in tZR transport between CCCP-treated non-induced and induced cells was lower when compared with non-treated cells (the median of *I* increased from 0.85×10^−3^ to 2.05×10^−3^ s^−1^ upon induction of *AtENT3* expression in CCCP-treated cells), but it remained statistically significant. In NBTI-treated cells there was no significant difference between the control and induced cells, indicating strong inhibition of AtENT3 by NBTI. Finally, in DiPy-treated cells, the median of *I* mildly increased from 7.73×10^−3^ to 9.39×10^−3^ s^−1^, but the independent statistical test results deemed this increase non-significant; we thus interpret DiPy as an effective inhibitor of tZR uptake, possibly less potent than NBTI. The complete list of kinetic parameters and their statistical analysis is presented in Supplementary Tables S15 and S16, respectively. The results of the competition assays show that AtENT3 is inhibited by NBTI and (to a lesser extent) DiPy, two typical inhibitors of adenosine uptake. CCCP, an agent uncoupling the proton gradient, appears to weaken but not completely abolish the AtENT3-mediated transport of CKs. This observation suggests that the AtENT3 activity might be boosted by the proton gradient, while not fully depending on it.

### Plant-specific amino acids Tyr61 and Asp129 of AtENT3 are necessary for the cytokinin riboside transport function

**Fig. 5.**
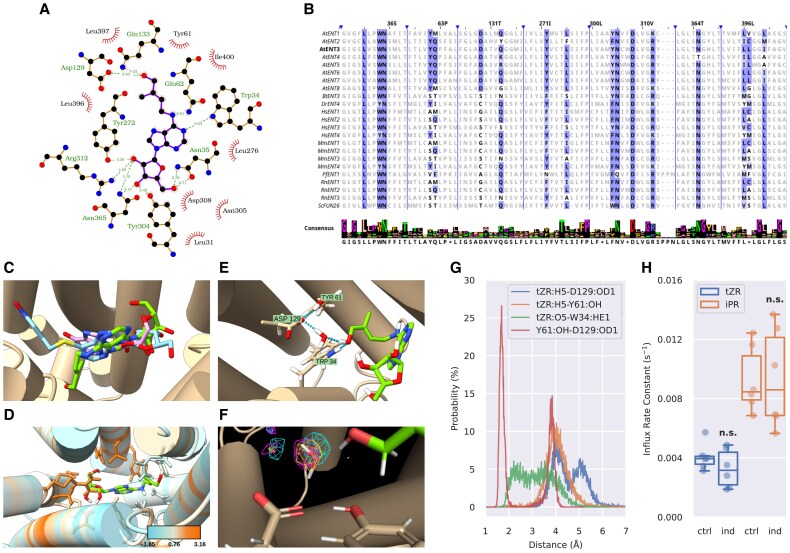
Computational assessment of the interactions between the AlphaFold-predicted structure of AtENT3 and tZR. (A) Schematic representation of the best docked pose of tZR in the binding cavity of AtENT3. Dashed lines represent hydrogen bonds with lengths given in Å. Short rays represent hydrophobic interactions. Visualized in LigPlot+. (B) Sequential alignment of plant, animal, parasitic, and yeast ENTs. The residues of AtENT3 interacting with the docked pose of tZR and their homologs are shown in bold. Their conservation among the presented species is depicted by the differential intensity of the highlight. Vertical lines mark breaks in the sequences. The labels in the header of the alignment denote the residues of AtENT3 found at the given position. The consensus sequences and the logotype of the alignment segments are given at the bottom. Visualized in Jalview. At, Arabidopsis; Bt, cattle (*Bos taurus*); Dr, zebrafish (*Danio rerio*); Hs, human (*Homo sapiens*); Mm, mouse (*Mus musculus*); Pf, malaria parasite (*Plasmodium falciparum*); Rn, rat (*Ratus norvegicus*); Sc, yeast (*Saccharomyces cerevisiae*). (C) Superimposition of AtENT3 (tan cylinders) with the docked pose of tZR (green) and the experimental poses of NBTI (light blue) in HsENT1 (PDB code: 6OB6) and inosine (pink) in PfENT1 (PDB code: 7WN1). The amino acid residues of 6OB6 and 7WN1 are hidden. (D) Sequence conservation of AtENT3 residues interacting with the docked pose of tZR calculated by the AL2CO program (Pei and Grishin, 2001) from the alignment depicted in (B). Larger numbers indicate higher conservation. (E) Hydrogen bonding among tZR, Trp34, Tyr61, and Asp129, and a water molecule in the system equilibrated by molecular dynamics (MD). Hydrogen bonds are depicted as dashed lines. Images (C–E) are visualized in UCSF ChimeraX. (F) Fractional occupancies of the MD simulation grid by water molecules. The meshes represent isosurfaces with the fractional occupancy of 90%. Differently coloured meshes correspond to three independent MD simulation runs. Visualized in PyMol. (G) Representative distributions of atomic distances involving the hydrogen (H5) and oxygen (O5) of the side-chain carboxyl of tZR, a carboxylic oxygen of Asp129 (OD1), the phenolic oxygen of Tyr61 (OH), and the nitrogen-bound hydrogen of Trp34 (HE1). (H) Influx rate constants characterizing uptake of radio-labelled tZR in BY-2 cells expressing a mutated version of *AtENT3* with Tyr61 and Asp129 replaced with serine. The mutated gene was introduced under an estradiol-inducible promoter into control (ctr) or estradiol-treated cells (ind). *P*-values obtained from the Kruskal–Wallis test comparing control and induced cells: n.s., *P>*0.05. ENT, EQULIBRATIVE NUCLEOSIDE TRANSPORTER; iPR, isopentenyladenosine; tZR, *trans*-zeatin riboside.

To assess the molecular interactions responsible for CK binding to AtENT3, we performed molecular docking of tZR into a predicted structure of AtENT3 obtained with AlphaFold (Jumper *et al.*, 2021). The best-docked pose of tZR was located in a central cavity outlined by transmembrane helices (TMs) 1, 2, 4, 5, 7, 8, 10, and 11. The ENT3 residues interacting with the docked pose of tZR comprised Leu31, Trp34, Asn35, Tyr61, Gln62, Asp129, Gln133, Tyr272, Leu276, Tyr304, Asn305, Asp308, Arg312, Asn365, Leu396, Leu397, and Ile400 (Fig. 5A). This pose roughly corresponds to the sites occupied by the adenosyl moiety of NBTI in human HsENT1 (Wright and Lee, 2019) (PDB code: 6OB6) and by inosine in PfENT1 from the parasite *Plasmodium falciparum* (Wang *et al.*, 2023) (PDB code: 7WN1; Fig. 5C). Moreover, the CK-interacting residues Leu31, Trp34, Gln133, Tyr304, Asp308, and Lys312 correspond to residues reported to bind respective ligands in 6OB6 and 7WN1. We also performed docking of iPR, tZ, and iP. Their best-docked poses are shown in Supplementary Figs S2–S4. In comparison with tZR, the best-scoring docked pose of the CK nucleobase tZ lacks interactions with Asn35, Tyr272, Tyr304, Asn305, Asp308, Arg312, and Asn365. These amino acids are predicted to bind the ribosyl moiety of tZR (Fig. 5A), which makes their lack of interactions with tZ understandable. Conversely, the best-scoring docked pose of tZ in addition interacts with Thr58, Leu38, Pro280, and Val404, which are not predicted to bind tZR (and with an exception of Leu38, neither iPR).

To assess the conservation of the CK-binding residues among the known members of the ENT family, we aligned sequences of the reviewed ENT proteins present in the UniProtKB database (The UniProt Consortium, 2023). These proteins are AtENT1-8, BtENT3 from cattle, DrENT4 from zebrafish, HsENT1-4, MmENT1-4 from mouse, PfENT1 from malaria parasite, RnENT1-3 from rat, and ScFUN26 from yeast. The alignment (Fig. 5B) shows that the residues interacting with the ribosyl moiety of tZR are generally more conserved than those interacting with the heterocycle and the side chain (Fig. 5D), suggesting that the binding cavities of different ENTs are all shaped to recognize nucleosides but with different specificities towards various aglycones.

To estimate the stability of predicted AtENT3-tZR interactions, we performed molecular dynamic simulations with a system consisting of the AtENT3-tZR complex in water and 150 mM NaCl. To equilibrate the system, we ran a single 200 ns-long simulation. Through cluster analysis of the 200 ns-long trajectory, we obtained a representative system conformation (corresponding to the frame at *t* = 177.72 ns). In this conformation, we observed interactions between the side-chain hydroxyl group of tZR and residues Trp34, Tyr61, and Asp129, mediated by a water molecule (Fig. 5E). Tyr61 and Asp129 are conserved among AtENTs but not among ENTs from other species listed in Fig. 5B, suggesting they might have a unique role in binding CK substrates.

Next, we ran three parallel 100 ns-long simulations, starting from the system conformation obtained through the cluster analysis. In each simulation we measured distances between atoms that mediate the interactions of tZR with Trp34, Tyr61, and Asp129. Histograms of tZR-Tyr61 and tZR-Asp129 distances showed sharp peaks, indicating stable interactions. Similarly, the histogram distances between Tyr61 and Asp129 showed that the amino acids strongly interact with one another, too. All histograms involving Asp129 also displayed a secondary peak, which can be explained by flipping of the Asp129 carboxyl group. Conversely, there was no sharp peak in the histogram of distances between tZR and Trp34, indicating that their interaction is not stable (Fig. 5G).

The peaks of tZR-Tyr61 and tZR-Asp129 distance histograms lie approximately at 4.0 Å, which is longer than the typical hydrogen bond (Lemkul, 2019). A possible explanation of this observation is that the interaction between tZR and the amino acids is not direct but rather stably mediated by a water molecule, as shown in Fig. 5E. To confirm this, we measured distribution of water molecules throughout the system across all three 100 ns-long simulations. The space surrounded by the side chains of tZR, Tyr61, and 129 was occupied by a water molecule in more than 90% of the total simulation time in all three simulations (Fig. 5F), which supports the involvement of water in maintaining tZR-Tyr61 and tZR-Asp129 interactions.

Having selected Tyr61 and Asp129, we tested their involvement in the binding of CKs by preparing a mutated version of *AtENT3* with both Tyr61 and Asp129 replaced by serine. We introduced the estradiol-inducible *AtENT3:Y61S:D129S:GFP* construct bearing these two mutations into BY-2 cells and checked its expression microscopically (Supplementary Fig. S5). Then, we repeated the radio-accumulation assays with [^3^H]tZR and [^3^H]iPR in BY-2 cells expressing the site-mutated construct. Induction of the mutated version of *AtENT3* did not increase accumulation of either [^3^H]tZR or [^3^H]iPR at all (Fig. 5H). The estimated values of kinetic parameters and their statistical analysis is presented in Supplementary Tables S17 and S18, respectively. Combining computational and experimental approach, we showed that plant-specific residues Tyr61 and Asp129 are necessary for recognition of CKs as substrates of AtENT3. It is worth noticing that both Tyr61 and Asp129 also bind the best-scoring docked poses of iPR, tZ, and iP (Supplementary Fig. S2–S4), suggesting they might be important for CK binding in general.

### Loss of the AtENT3 function affects shoot development

We went on to assess the effects of the disrupted transport of riobsylated CKs in plants. We started by examining the phenotypes of Arabidopsis plants mutated in or overexpressing the *AtENT3* gene. Given the implication that root-borne CK ribosides are crucial for CK-mediated processes in the shoot apex (Sakakibara, 2021), and the fact that the effects of *atent3* mutation on the primary root length have been reported recently (Korobova *et al.*, 2021), we focused on the phenotype of shoots.

**Fig. 6.**
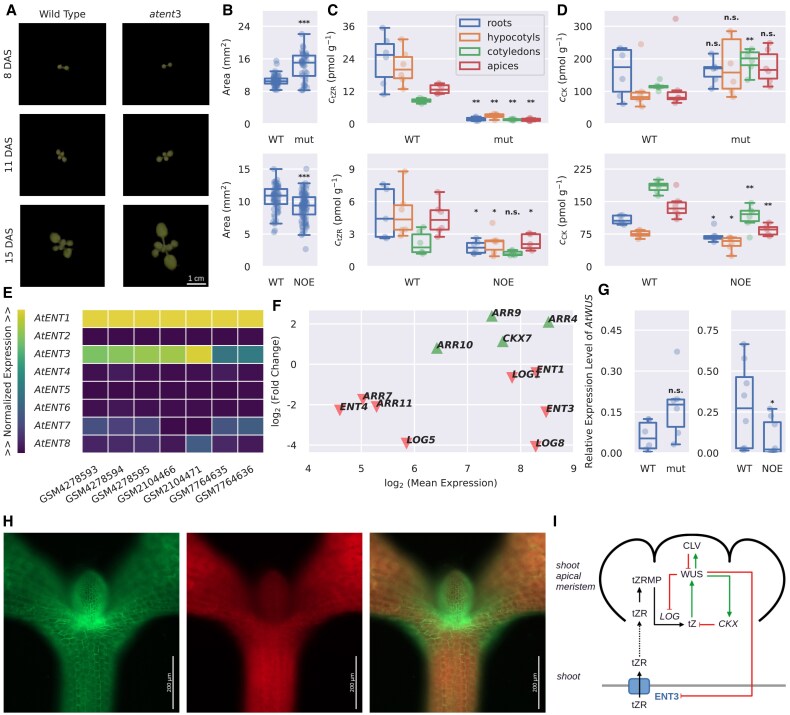
AtENT3-mediated transport of tZR contributes to the shoot development in Arabidopsis. (A) Top view images of wild-type and *atent3* plants grown in pots. (B) Shoot areas of 8-day-old *atent3* (top) and *AtENT3*-overexpressing plants (bottom) grown on ^1^/_2_ MS medium measured through image analysis. (C) tZR concentrations in roots, hypocotyls, cotyledons, and apices of 8-day-old *atent3* (top) and *AtENT3*-overexpressing plants (bottom) plants. The legend also applies to plot (D). (D) Total CK concentrations in roots, hypocotyls, cotyledons, and apices of 8-day-old *atent3* (top) and *AtENT3*-overexpressing plants (bottom) plants. (E) Relative expressions of *AtENT* genes in the shoot apices or apex-enriched tissues, retrieved from the Gene Expression Ominubs (GEO) through the listed accessions. The colour scale is normalized from 0 to the maximal value in each column. (F) Differential expression of genes related to CK transport, metabolism, and signalling in Arabidopsis plants ectopically overexpressing *AtWUS* in comparison with control plants. Data obtained from GEO accession GSE122610. (G) Relative expression levels of *AtWUS* in the shoots of 8-day-old *atent3* (left) and *AtENT3*-overexpressing plants (right) obtained through quantitative PCR. (H) *pAtENT3::GFP:AtENT3* expression pattern in 4-day-old Arabidopsis shoots. Green fluorescent protein (GFP) fluorescence at 475 nm (left), chlorophyll autofluorescence at 630 nm (middle) and their overlay (right) are shown. (I) A schematic depiction of the function of AtENT3-mediated tZR transport in the maintenance of CK homeostasis in shoots and AtWUS activity in the shoot apical meristem. Black arrows denote movement and conversions of cytokinin species, green arrows activation, and red lines with flat ends inhibition. *P*-values obtained from the Kruskal–Wallis test comparing wild-type plants with *atent3* or *AtENT3* native overexpressors: n.s., *P*≥0.05; *P<0.05, **P<0.01, ***P<0.001. ARR, ARABIDOPSIS RESPONSE REGULATOR; CK, cytokinin; CKX, CYTOKININ DEHYDROGENASE; CLV, CLAVATA; DAS, days after sowing; mut, *atent3* mutant; ENT, EQULIBRATIVE NUCLEOSIDE TRANSPORTER; LOG, LONELY GUY; NOE, *AtENT3* native overexpressor; tZR, *trans*-zeatin riboside; tZRMP, *trans*-zeatin riboside monophosphate; WT, wild type.

We imaged shoots of 8-day-old wild-type plants*, atent3* mutants, and native *AtENT3* overexpressors grown on the ^1^/_2_ MS medium, as well as the shoots of 8-, 11-, and 15-day-old wild-type and *atent3* plants grown on the cultivation substrate in pots. We processed the obtained image to measure the area of plant shoots from top view. The shoots of *atent3* plants were larger than the corresponding control in all cases (Fig. 6A, B), and conversely, the shoots of *AtENT3* overexpressors were smaller (Fig. 6E). All measured parameters are presented in Supplementary Tables S19 and S20. Statistical analysis of measured areas is presented in Supplementary Table S21. To see whether the observed changes in shoot size are to be attributed to altered CK signalling, we measured CK concentrations in the roots, hypocotyls, cotyledons, and apices of 8-day-old wild-type, *atent3*, and *AtENT3*-overexpressing plants. Concentrations of tZR, the main xylem-transported CK form, was decreased in all listed tissues of *atent3* plants, as well as in the roots, hypocotyls, and apices of *AtENT3* overexpressors compared with the respective controls (Fig. 6C), suggesting that both mutation and overexpression of *AtENT3* disrupt the long-distance transport of tZR. Conversely, the summed concentration of all CKs was increased in the *atent3* cotyledons (the median concentrations were also increased in *atent3* hypocotyls and apices, but the statistical significances from the Kruskal–Wallis test for these differences were borderline) but decreased in all *AtENT3*-overexpressing tissues (Fig. 6D). These results indicate that AtENT3 is also involved in maintaining local CK homeostasis, especially in shoots. Concentrations of all detected CK species and their statistical analyses are presented in Supplementary Tables S22 and S23, respectively.

The effect of AtENT3 on the CK homeostasis in shoots indicates that AtENT3 might be involved in supplying the shoot apex with tZR and thus co-regulate developmental processes in the shoot apical meristem driven by WUSCHEL (WUS) (Sakakibara, 2021). This hypothesis assumes *AtENT3* expression in the shoot apex vicinity. We therefore imaged the expression pattern of the *pAtENT3::GFP:AtENT3:3′-UTR* construct (expressing the *GFP:AtENT3* fusion under the native *AtENT3* promoter) in 4-day-old seedlings. As shown in Fig. 6H, *AtENT3* expression was indeed enriched close to the shoot apex, namely at the bases of cotyledons and first true leaves. We also examined gene expression data from isolated apices or apex-enriched tissues of Arabidopsis deposited to the Gene Expression Omnibus (GEO) database under accessions GSM4278593-95 (Yang *et al.*, 2021), GSM2104466 and 71 (Mandel *et al.*, 2016), and GSM7764635-36 (Incarbone *et al.*, 2023). From these data, we extracted expression levels of *AtENT1-8*. In all samples, *AtENT1* and *3* were abundantly expressed, sometimes followed by *AtENT7* and *8* (Fig. 6E), supporting the idea that AtENT3 supplies the shoot apex with root-borne tZR.

We also examined gene expression data from 5-day-old ectopic *AtWUS* overexpressors deposited in the GEO database under the accession GSE122610 (Ma *et al.*, 2019). In this dataset we looked for members of the *AtENT* family and genes related to CK metabolism, transport or signalling. Overexpression of *AtWUS* down-regulates *AtENT3* and *4*; CK-activating genes from the LONELY GUY (LOG) family, *AtLOG5*, *6* and *8*; and CK-responsive genes from the ARABIDOPSIS RESPONSE REGULATOR (ARR) family, *ARR7* and *11*. Conversely, *ARR4*, *9*, and the CK-degrading CYTOKININ DEHYDROGENASE 7 (*AtCKX7*) were up-regulated (Fig. 6F). Overall, overexpression of *WUS* leads to a decrease in the CK pool, suggesting a negative feedback mechanism depicted schematically in Fig. 6I.

To see whether alternations in AtENT3-mediated transport of CK ribosides impact AtWUS (and thus whether AtENT3 acts upstream of the AtWUS-mediated processes), we measured *AtWUS* relative expression levels, defined by Equation (4), in the shoots of wild-type, *atent3*, and *AtENT3*-overexpressing plants. The measured values of the relative expression levels of *AtWUS* are depicted in Fig. 6G. In the shoots of *AtENT3* overexpressors the expression of *AtWUS* was decreased, which can be related to the decrease of tZR concentrations in the entire plants, as well as to the decrease of total CKs in the *AtENT3-*overexpressing shoots. In *atent3* mutants the median of the relative expression level of *AtWUS* increased, but the *P*-value obtained from the Kruskal–Wallis test was above the chosen threshold level of 0.05, so we interpret this increase as non-significant. The numerical values of the relative expression levels of *AtWUS* and their statistical analysis are given in Supplementary Tables S24 and S25, respectively.

## Discussion

Ribosylated CKs are the dominant CK form transported through the xylem and phloem (Takei *et al.*, 2001; Corbesier *et al.*, 2003; Sakakibara, 2021). Their effective distribution between the cellular and extracellular compartments, mediated by membrane-bound carriers, is thus a crucial aspect of communication among different tissues and organs. In this work, we address particular differences between the transport kinetics of CK nucleobases and ribosides via radio-accumulation assays in the BY-2 cell culture, a plant model system. We show that the uptake kinetics of CKs with isoprenoid chains differ significantly more between nucleobases and ribosides than among compounds with different side chain compositions, highlighting the presence of CK riboside-specific transporters (Fig. 1). Conversely, the uptake kinetics of aromatic CKs (BA and BAR) do not differ, suggesting that aromatic CKs are recognized by a different transport system. The specific transport properties of BA and BAR evoke inquiries about the roles of aromatic CKs in plants and the means of maintaining their homeostasis in general.

We further show that the CK uptake occurs via at least two different systems of membrane-bound carriers. One of these systems exclusively recognizes ribosylated substrates. The other one primarily transports nucleobases but not as strictly as the first system. We derive this conclusion from the general trend of our data from accumulation assays, which shows that the inhibition of the CK riboside uptake by nucleobases is weaker than when the roles are reversed (Fig. 3). The existence of riboside-specific transporters hypothetically allows plants to regulate the distribution of ribosylated CKs in a targeted manner. Since ribosylated CKs are primarily transported over long distances, the CK riboside-specific carriers could be found within or close to vascular tissues. This particular expression pattern could serve as a clue in the search for other CK riboside transporters in future research.

We used AtENT3 as a representative membrane-bound carrier of CK ribosides to further characterize the CK riboside transport. Our measurements of CK uptake in the *AtENT3*-expressing BY-2 cells showed that AtENT3 transports CK nucleobases, as well as ribosides. Thus, AtENT3 does not belong among the CK riboside-specific carriers (Fig. 4). Similar measurements focused on the ability of other AtENTs to distinguish between CK nucleobases and ribosides would allow us to tell whether this trend observed for AtENT3 applies to all AtENTs or whether the family includes other members that may be specific for CK ribosides. The molecular docking of tZ and iP into an AlphaFold-predicted structure of AtENT3 (Supplementary Figs S2, S3) shows that both nucleobases interact with Gln62 via a hydrogen bond. Gln62 is conserved among AtENT2–7, while in AtENT1 and 8 the corresponding position is occupied by methionine, an amino acid with an aliphatic side chain that is unlikely to form the mentioned hydrogen bond (Fig. 5B). We propose that due to this difference in the amino acid composition, AtENT1 and 8 will not recognize CK nucleobases as substrates, which will be interesting to prove or disprove in future experiments.

The character of the position corresponding to Gln62 in AtENT3 also varies among other ENTs listed in Fig. 5B. Notably, this position differs between HsENT1 (methionine) and HsENT2 (glutamine), consistent with their previously reported affinities towards nucleobases and nucleosides. HsENT1 favours uridine, a riboside, over nucleobases adenine, thymine, and hypoxanthine, whereas HsENT2 transports the nucleobases with affinities equal to that towards uridine or greater (Yao *et al.*, 2011). The differential affinity towards nucleobase substrates between HsENT1 and 2 supports the hypothesis that the variable nature of the position corresponding to Gln62 in AtENT3 can affect the substrate specificities of ENTs. Another example is PfENT1, which has the position corresponding to Gln62 in AtENT3 occupied by glutamate and transports inosine, a riboside, and hypoxanthine with comparable affinities (Wang *et al.*, 2023).

In the last part of this work, we have addressed the physiological impact of the AtENT3-mediated transport in Arabidopsis shoots. The membrane transport of CK ribosides has been deemed a necessary step for the activation of tZR coming via xylem from roots up to the shoot apex and subsequent stimulation of *AtWUS* expression (Davière and Achard, 2013; Osugi *et al.*, 2017; Landrein *et al.*, 2018; Lopes *et al.*, 2021; Sakakibara, 2021). This fact prompted us to investigate whether a change in *AtENT3* expression affects shoot development. We report that the shoots of *atent3* and *AtENT3*-overexpressing plants are larger and smaller compared with the controls, respectively (Fig. 6B). Considering that CKs generally promote shoot development (Werner *et al.*, 2003; Riefler *et al.*, 2006; Wybouw and De Rybel, 2019), larger shoots are to be associated with CK abundance, which corresponds to the overall increase of CK concentrations in the upper parts of *atent3* plants. Similarly, the smaller shoots of *AtENT3* overexpressors correspond to the decrease of total CK concentrations (Fig. 6D). These complementary effects of *atent3* mutation and *AtENT3* overexpression are, however, in stark contrast to the decreased concentration of tZR, the main CK form transported over long distances, in all *atent3* and *AtENT3*-overexpressing tissues (Fig. 6C). The drop in tZR concentrations in the whole plants suggests that both insufficient and exaggerated AtENT3 functionality disrupts the tZR-mediated communication between roots and shoots. This lack of communication possibly results in either enhanced or suppressed CK production in shoot tissues, suggesting that root-borne CKs act as regulators of local CK synthesis in the aerial parts and that AtENT3 is also involved in the short-distance distribution of these locally synthesized CKs. We support the latter by showing that *AtENT3* is both expressed and localized in the shoot tissues (Fig. 6E, H). The diverse effects of *AtENT3* expression on locally synthesized shoot CKs and root-borne tZR could be related to the fact that AtENT3 is found in both the shoots and the roots (Li *et al.*, 2003; Traub *et al.*, 2007; Cornelius *et al.*, 2012; Korobova *et al.*, 2021), implying that the relation between AtENT3 and the overall CK homeostasis is not monotonous.

We have been able to report only a limited effect of a change in *AtENT3* expression on the expression of *AtWUS*. Nevertheless, publicly available transcriptomic data revealed that the overexpression of *AtWUS* down-regulates the expression of *AtENT3*, as well as that of *AtENT4*, *AtLOG5*, *AtLOG6*, and *AtLOG8* (and to a lesser extent also that of *AtENT1* and *AtLOG1*). Conversely, *AtWUS* overexpression up-regulates *AtCKX7* (Ma *et al.*, 2019), altogether indicating that the overexpressed *AtWUS* tends to inhibit CK signalling in a form of negative feedback. This feedback likely occurs via activation of type-A ARRs, which are typically characterized as CK-repressive (To *et al.*, 2004, 2007). In the transcriptomic dataset by Ma *et al*. (2019) the overexpression of *AtWUS* up-regulates type-A *ARR4* and *ARR9*. On the other hand, type-A *ARR7* is down-regulated, consistently with a previous report on ARR5, 6, 7, and 15 acting downstream of AtWUS (Leibfried *et al.*, 2005). The differential effect of the *AtWUS* overexpression on the type-A ARRs could signify that plants distinguish between CK signalling over long distances (which modulates AtWUS activity in response to environmental cues) and CK signalling at the local level (which further shapes the shoot apex) by employing different response regulators for each. Establishing the relations between root-borne tZR and shoot-produced CKs is a prospective topic for future research, which might not only help to better understand the root-to-shoot communication but also uncover whether and how plants can discriminate between chemically related molecules based on their site of origin.

## Supplementary Material

eraf369_Supplementary_Data

## Data Availability

Additional raw data not included in the supplementary files, as well as step-by-step derivations of the mathematical models used in this work, are available at Zenodo (Nedvěd *et al.*, 2025). https://doi.org/10.5281/zenodo.16568211

## Supplementary data

The following supplementary data are available at *JXB* online.

**Fig. S1.** Expression pattern of the estradiol-inducible *AtENT3:GFP* construct in 2-day-old BY-2 cells used for radio-accumulation transport experiments.

**Figs. S2.** The best-docked position of tZ in the AlphFold-predicted structure of AtENT3.

**Figs. S3.** The best-docked position of iP in the AlphFold-predicted structure of AtENT3.

**Figs. S4.** The best-docked position of iPR in the AlphFold-predicted structure of AtENT3.

**Fig. S5.** Expression pattern of the estradiol-inducible *AtENT3:Y61S:D129S:GFP* construct in 2-day-old BY-2 cells used for radio-accumulation transport experiments.

**Table S1.** Parameters for molecular dynamic simulations.

**Table S2.** Sequences of primers used for cloning and quantitative PCR.

**Table S3.** Kinetic parameters obtained through the mathematical modelling of accumulations assays with radio-labelled tZ, tZR, iP, iPR, BA, BAR, DHZ, and DHZR in BY-2 cells.

**Table S4.** Statistical analysis of influx rate constant data from Supplementary Table S3.

**Table S5.** Purity of applied radio-labelled cytokinin tracers.

**Table S6.** Metabolic conversions of radio-labelled cytokinins in BY-2 cells.

**Table S7.** Kinetic parameters obtained through the mathematical modelling of accumulation assays with radio-labelled tZ, tZR, iP, and iPR and their non-labelled counterparts at various concentrations as inhibitors.

**Table S8.** Kinetic parameters obtained through the mathematical modelling of accumulation assays with radio-labelled tZ, tZR, iP, and iPR with or without the addition of 10 µM CCCP at the beginning of the assay.

**Table S9.** Statistical analysis of influx rate constant data from Supplementary Table S8.

**Table S10.** Kinetic parameters obtained through the mathematical modelling of accumulation assays with radio-labelled tZ, tZR, iP, and iPR with the addition of 10 µM CCCP at *t*=420 s.

**Table S11.** Kinetic parameters obtained through the mathematical modelling of accumulation assays with radio-labelled tZ, tZR, iP, iPR, BA, and BAR and various nucleobases and ribosides at the concentration of 20 µM as competitors.

**Table S12.** Expression levels of tobacco *ENT* homologs in BY-2 cells. Data taken from GSE160438.

**Table S13.** Kinetic parameters obtained through the mathematical modelling of accumulation assays with radio-labelled tZ, tZR, iP, and iPR in control and induced BY-2 cells harbouring the *AtENT3* gene.

**Table S14.** Statistical analysis of influx rate constant data from Supplementary Table S13.

**Table S15.** Kinetic parameters obtained through the mathematical modelling of accumulation assays with radio-labelled tZR and NBTI, Dipy or CCCP as inhibitors in control and induced BY-2 cells harbouring the *AtENT3* gene.

**Table S16.** Statistical analysis of influx rate constant data from Supplementary Table S15.

**Table S17.** Kinetic parameters obtained through the mathematical modelling of accumulation assays with radio-labelled tZR and iPR in control and induced BY-2 cells harbouring the *AtENT3* gene with Tyr61 and Asp129 replaced with serine.

**Table S18.** Statistical analysis of influx rate constant data from Supplementary Table S17.

**Table S19.** Parameters obtained through processing of images of 8-day-old wild-type, *atent3*, and *AtENT3*-overexpressing Arabidopsis plants grown on the agar.

**Table S20.** Parameters obtained through processing of images of 8, 11, and 15-day-old wild-type and *atent3* Arabidopsis plants grown in pots.

**Table S21.** Statistical analysis of shoot area data from Supplementary Tables S19, S20.

**Table S22.** Cytokinin concentrations in shoot apices, cotyledons, hypocotyls, and roots of wild-type, *atent3*, and *AtENT3-*overexpressing Arabidopsis plants.

**Table S23.** Statistical analysis of tZR and total CK concentrations from Supplementary Table S22.

**Table S24.** Relative expression levels of *AtWUS* measured through quantitative PCR in the shoots of 8-day-old wild-type, *atent3*, and *AtENT3-*overexpressing Arabidopsis plants.

**Table S25.** Statistical analysis of data from Supplementary Table S24.

## Abbreviations

ARR: ARABIDOPSIS RESPONSE REGULATOR
BA: benzyladenine
BAR: benzyladenosine
CCCP: carbonyl cyanide 3-chlorophenylhydrazone
CK: cytokinin
CKX: CYTOKININ DEHYDROGENASE
DHZ: dihydrozeatin
DHZR: dihydrozeatin riboside
DiPy: dipyridamole
DMSO: dimethyl sulfoxide
ENT: EQULIBRATIVE NUCLEOSIDE TRANSPORTER
iP: isopentenyladenine
iPR: isopentenyladenosine
LOG: LONELY GUY
NBTI: 4-nitrobenzylthioinosine
NOE: native overexpressor
WUS: WUSCHEL
tZ: *trans*-zeatin
tZR: *trans*-zeatin riboside
