# Supplementary material for: Membrane transport of root-borne trans-zeatin riboside maintains the cytokinin homeostasis in shoots
Source: J Exp Bot. 2025 Aug 25;76(22):6723–40. doi: 10.1093/jxb/eraf369 (PMC12675277; doi:10.1093/jxb/eraf369)
Supplement: eraf369_Supplementary_Data [file eraf369_supplementary_data.zip › jexbot315894-file001.pdf]

1 Supplementary Figures

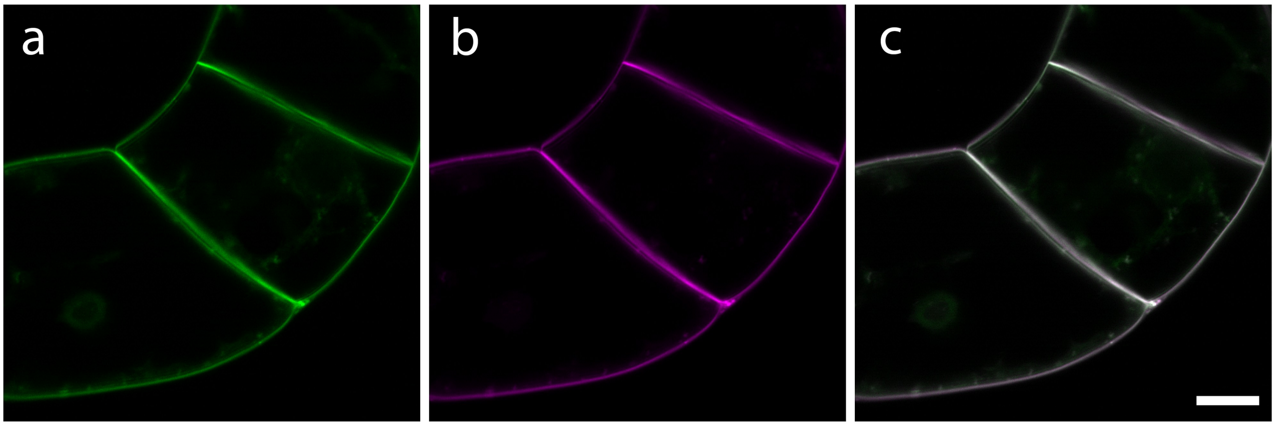

Figure S1: Expression pattern of the estradiol-inducible AtENT3:GFP construct in 2-day-old BY-2 cells used for radio-accumulation transport experiments. a) AtENT3:GFP, b) incubation with the membrane-probing FM 4-64 dye (2 µM, 20 minutes), c) merged signals showing co-localization on the plasma membrane. Scale bar = 10 µm.

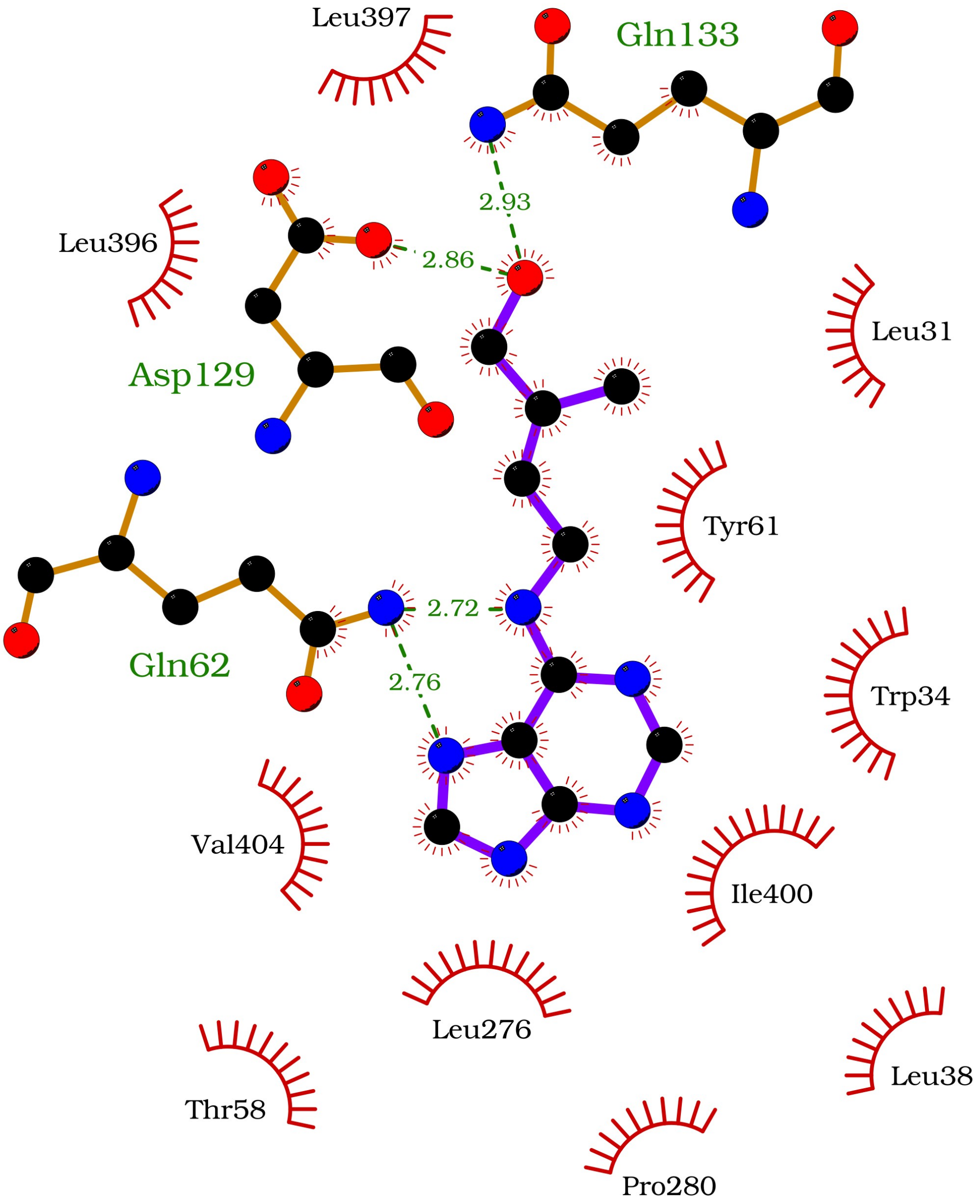

Figure S2: The best-docked pose of trans-zeatin in the AlphaFold-predicted strucutre of AtENT3. Visuazlied in LigPlot+.

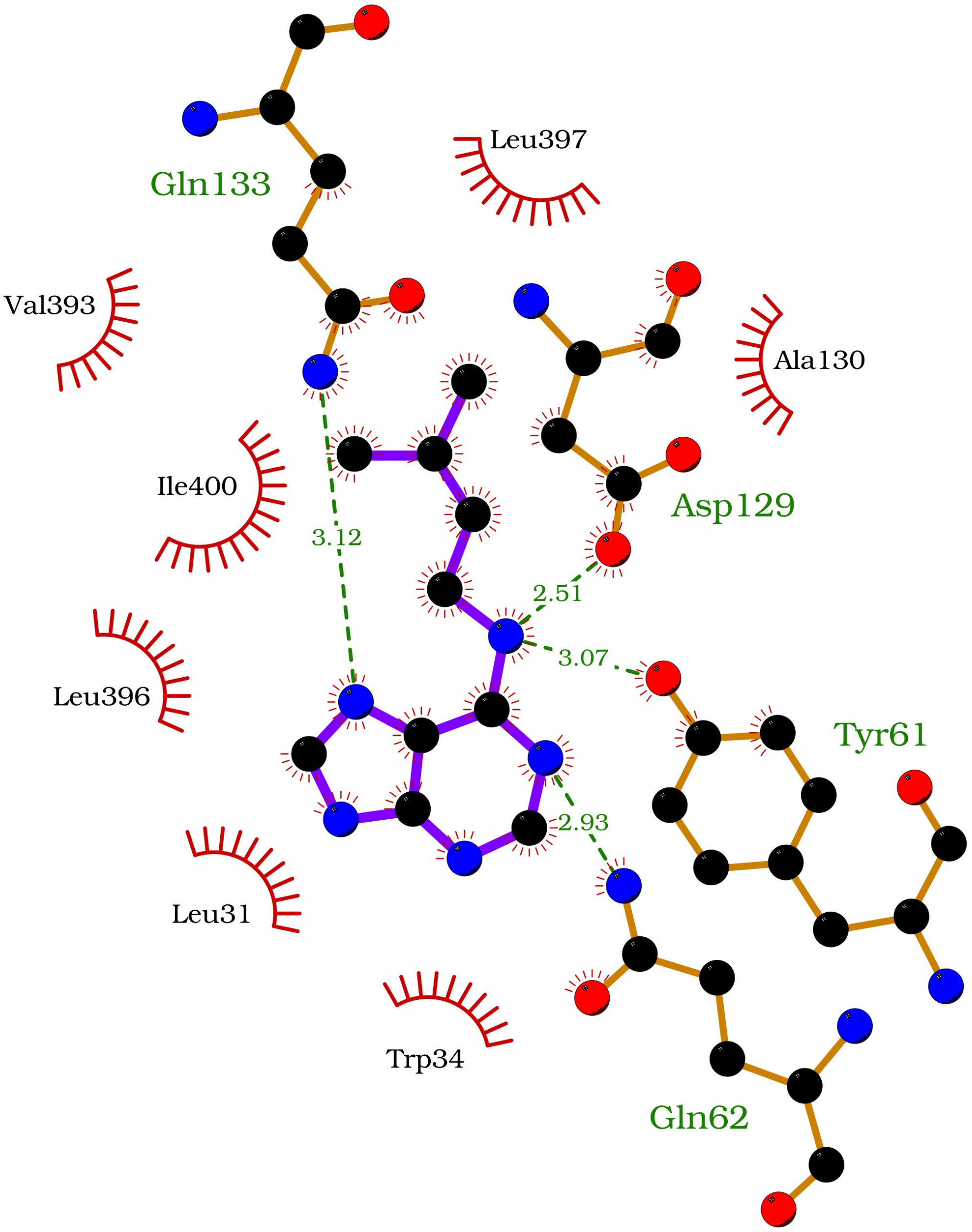

Figure S3: The best-docked pose of isopentenyladenine in the AlphaFold-predicted strucutre of AtENT3. Visuazlied in LigPlot+.

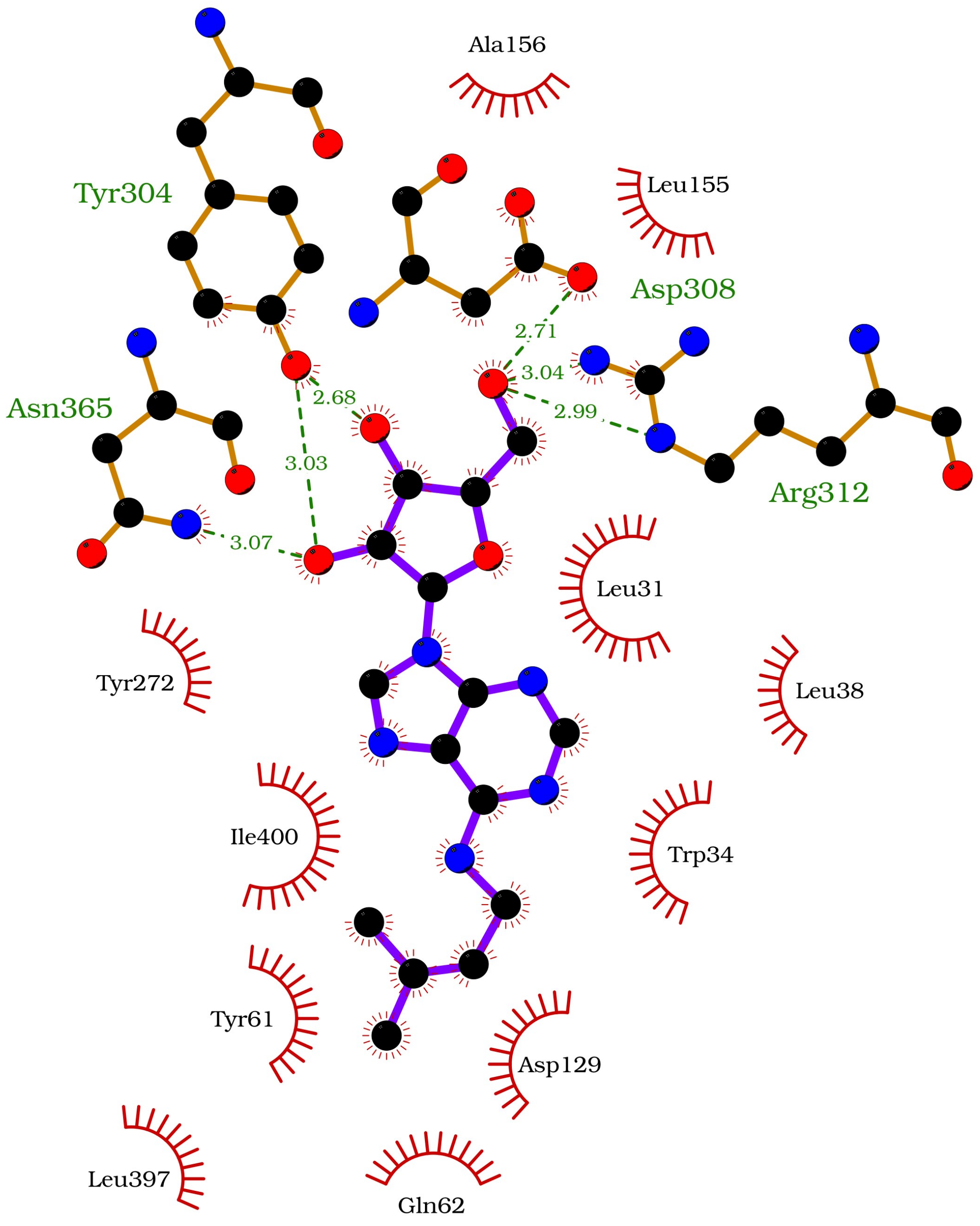

Figure S4: The best-docked pose of isopentenyladenosine in the AlphaFold-predicted strucutre of AtENT3. Visuazlied in LigPlot+.

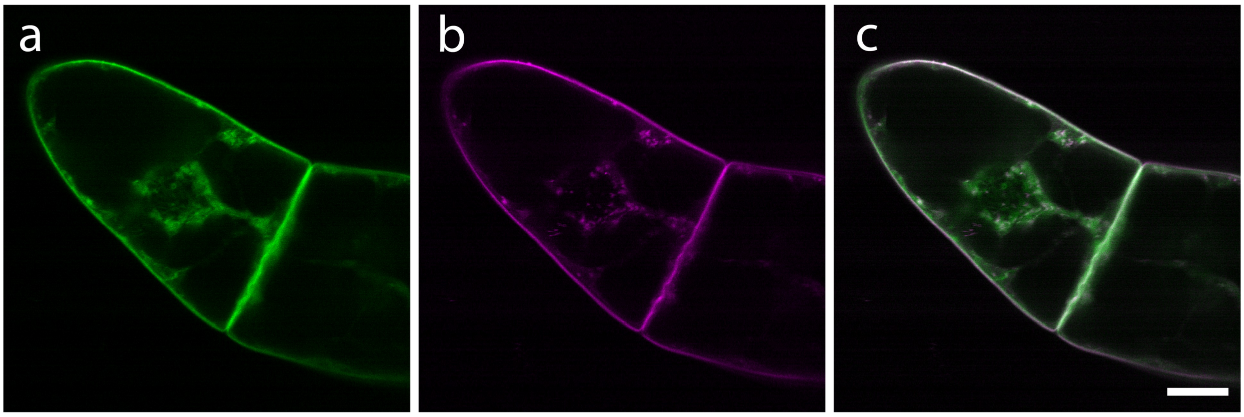

Figure S5: Expression pattern of the estradiol-inducible AtENT3:Y61S:D129S:GFP construct in 2-day-old BY-2 cells used for radio-accumulation transport experiments. a) AtENT3:Y61S:D129S:GFP, b) FM 4-64 (2 µM, 20 minutes), c) merged signals showing co-localization on the plasma membrane. Scale bar = 10 µm.
